# Antipsychotics and other risk factors for mortality among people with schizophrenia during an extreme heat event: a population-based case-control study

**DOI:** 10.1038/s41598-025-17591-0

**Published:** 2025-10-03

**Authors:** Shirley X. Chen, Michael J. Lee, David A. McVea, Sarah B. Henderson

**Affiliations:** 1 Environmental Health Services, British Columbia Centre for Disease Control, Vancouver, Canada; 2 Public Health Agency of Canada, Ottawa, Canada

**Keywords:** Risk factors, Schizophrenia, Environmental health, Epidemiology

## Abstract

Schizophrenia is associated with increased mortality during extreme heat events (EHEs), including the unprecedented 2021 Western North America EHE in British Columbia, Canada. We sought to examine the association between mortality during the 2021 EHE and multiple risk factors among people with schizophrenia, with a focus on antipsychotic medications. We identified all individuals with schizophrenia in British Columbia using an administrative chronic disease registry and linked them with antipsychotic dispensations for the 30 days prior to the EHE. We compared 137 individuals who died during the EHE with 57,394 who survived using multiple logistic regression. Analyses also included age, sex, income assistance, comorbidities, and indicators of schizophrenia severity. Dispensation of any antipsychotic was associated with increased EHE mortality [OR 2.43, 95% CI 1.52, 4.01], which was driven by haloperidol, clozapine, and zuclopenthixol. The risk was increased for dispensation of two or more antipsychotics in combination [OR 4.05, 95% CI 2.41, 6.98]. Other variables associated with EHE mortality included being on income assistance, age, sex, having a mental health-related emergency department visit, and time since disease registry entry. Being dispensed an antipsychotic prior to the EHE was associated with increased odds of mortality among people with schizophrenia. Some antipsychotics had larger effects, and risk increased with combination therapy. There were also significant non-pharmaceutical risk factors. People with schizophrenia are at-risk during EHEs due to multiple overlapping factors, including antipsychotic medications.

**Supplementary Information:**

The online version contains supplementary material available at 10.1038/s41598-025-17591-0.

## Introduction

Exposure to high ambient temperatures during extreme heat events (EHEs) can be associated with significant increases in population mortality. For example, more than 70,000 excess deaths across Europe were attributed to an extended EHE in the summer of 2003^1^ and there has been substantial excess mortality during many other events, including in Chicago, USA in 1995^2^, Moscow, Russia in 2010^3^, and Montreal, Canada in 2018^4^. The frequency, intensity, and duration of EHEs have all increased since the 1950s, which is projected to continue in the coming decades because of climate change^5^. Future mortality due to EHEs is expected to increase in the absence of health interventions and adaptation, particularly among susceptible populations^6^. Therefore, it is critical to understand why certain individuals experience heightened susceptibility to EHEs to develop targeted interventions for preventing heat-related injuries and deaths.

There is growing evidence that people with schizophrenia are at high risk during EHEs. For example, the rate of death among those with schizophrenia and related illnesses doubled during heat wave periods from 1993 to 2006 in Adelaide, Australia^7^. Similarly, during the 2018 event in Montreal, Canada, 25% of decedents had schizophrenic disorders despite a 0.6% prevalence of these conditions in the underlying population^4^. There are multiple and overlapping factors that may put people with schizophrenia at higher risk. First, many people with schizophrenia exhibit anosognosia, or lack of insight into their own health status^8^. This can affect their ability to perceive and respond to the threat of ambient heat. Second, schizophrenia may co-occur with other health conditions, including substance use disorder, hypertension, and diabetes, which are also risk factors for heat-related mortality^9,10^. Third, schizophrenia is associated with economic marginalization and social isolation^11^ which are also independent risk factors for heat-related mortality. Finally, antipsychotic medications can affect thermoregulation by inhibiting sweating and decreasing thirst^12^. While previous studies have identified schizophrenia as a risk factor for EHE mortality, none have directly investigated the individual characteristics of people with schizophrenia who died during a specific EHE^13^.

In the summer of 2021, western North America experienced a record-breaking EHE which was rapidly attributed to climate change^14^. During this event, population mortality across the province of British Columbia (BC), Canada increased by 95%, corresponding to 740 excess deaths^15^. As a part of a concerted effort to understand who was the most at risk, the BC Centre for Disease Control (BCCDC) first reported that the odds of death among people with schizophrenia tripled during the EHE when compared with deaths in previous years^16^. In this study, we aimed to further examine this association by focusing on the provincial population of people with schizophrenia. Our objective was to assess the association between mortality and antipsychotic drug dispensations as well as other potential risk factors including age, sex, income assistance, other comorbidities, and indicators of schizophrenia severity. To do this, we used an administrative chronic disease registry to identify all people in BC with schizophrenia, then compared those who died during the EHE with those who survived using a population-based case-control design.

## Methods

### Study context

The province of BC is in western Canada on the Pacific Ocean. The majority (60%) of its approximately 5.0 million residents live in the southwestern part of the province^17^. The climate is temperate, and a minority of homes have air conditioning, especially in the densely populated urban areas of greater Vancouver and Victoria^18^. During the 2021 EHE, record-breaking temperatures 16–20℃ above seasonal norms were recorded across much of the province over several days^14,19^. The town of Lytton, BC, set the all-time Canadian temperature high of 49.6 °C on June 29 before being destroyed by a wildfire the following day^19^.

The EHE was caused by a weather event known as an “omega block” or a “heat dome”, during which atmospheric high pressure prevents air movement allowing temperatures to build over consecutive days^14,20^. It occurred just after the summer solstice, when daylight lasts for 16–19 h south to north across the province, which contributed to high solar heat gain indoors. The EHE also contributed to high concentrations of ground level ozone (O_3_), especially in the greater Vancouver area where more than half of the deaths occurred^16^. Finally, the EHE occurred during the COVID-19 pandemic, at a time when many non-pharmaceutical interventions were still in place, and people were discouraged from gathering indoors. In this analysis we considered the overall impact of the EHE period, which included the effects of extreme heat, air pollution, and the societal context.

### Study design and population

We defined the study period as 25 June to 2 July 2021, corresponding to the period when significant excess mortality coincided with extreme temperatures as identified by our previous studies^16,21^. We used a population-based case-control study design to understand the association between EHE mortality, antipsychotic drug dispensations, and other risk factors among those with schizophrenia. All people living with schizophrenia in BC prior to the EHE were identified using an administrative chronic disease registry, which is described in the following section. Cases were defined as those with schizophrenia who died of any cause during the study period, and controls were defined as those with schizophrenia who survived for at least 30 days past the end of the study period. Individuals who entered the schizophrenia registry less than 30 days before the EHE were excluded from the analyses.

### Data

#### COVID-19 data library

The Provincial Health Services Authority and the BC Ministry of Health established the COVID-19 Data Library to inform rapid public health response during the COVID-19 pandemic^22^. This cloud-based data platform provides access to provincial administrative health records linkable at the individual level using a unique and anonymous patient identifier^22^. The Medical Services Plan (MSP) is the single-payer provincial public health insurance plan with mandatory enrolment for residents who live in the province for six months or longer. All residents in BC who are covered by MSP are captured in this data library, and all data described below were extracted from the library. The BCCDC received authorization from the BC Ministry of Health to use the COVID-19 Data Library to perform analyses related to the 2021 EHE as part of its mandate to generate evidence about the public health consequences of the EHE. As such, ethics approval was not required.

#### Chronic disease registries

The BC Ministry of Health maintains 26 administrative chronic disease registries (CDRs), which classify individuals as having a condition based on their patterns of healthcare usage. An individual enters and remains in the schizophrenia CDR in the event of one schizophrenia-related hospitalization or two schizophrenia-related MSP billings recorded more than 30 days apart within a two-year period. For chronic diseases that are relapsing-remitting such as schizophrenia, there is also an indicator for re-occurrence of the condition. We used the schizophrenia CDR to identify all individuals with schizophrenia, including their time since entry into the registry and whether they had a schizophrenia relapse in the year preceding the EHE. Among those with schizophrenia, we used other CDRs to identify those with diabetes, hypertension, and substance use disorder, each of which has its own publicly available inclusion criteria^23^. We examined these comorbidities because they frequently co-occur with schizophrenia^9,10^.

#### Antipsychotic dispensations

The PharmaNet database includes records on all prescription drugs dispensed by community pharmacies in BC^24^. Cases and controls were linked to records in PharmaNet to extract information on antipsychotic dispensations. Exposure was defined as the dispensation of at least one antipsychotic medication during the 30-day window before the EHE (26 May 2021–24 June 2021). We chose 30 days to capture the dispensation of long-acting injectable antipsychotics, which are most often administered monthly. As a follow-up analysis, we identified individuals who were continuously exposed in the year preceding the EHE. We defined continuous exposure as any combination of antipsychotic dispensations covering at least 75% (275 days) of the previous year.

Medications are classified into groups through the World Health Organization (WHO) Anatomical Therapeutic Chemical (ATC) Classification System based on the organs or systems they target and their therapeutic, pharmacological, and chemical properties^25^. We extracted all medications in the ATC group “N05A Antipsychotics”. Information included the type of antipsychotic medication by active ingredient, the number of days of supply, and whether the medication was dispensed in injectable form. Injectable antipsychotics last longer than oral medications and are often used in patients who have poor adherence to medication and may therefore be indicative of greater disease severity^26^.

Another potential indicator related to disease severity is the use of combination therapy, meaning concomitant treatment with two or more antipsychotic medications^27^. Combination therapy is often prescribed for schizophrenia patients due to treatment resistance, which occurs in 10–30% of cases^28^. We created a variable during the 30-day window with three levels of exposure: (1) no antipsychotic therapy (no antipsychotic dispensed); (2) monotherapy (one antipsychotic dispensed); and (3) combination therapy (two or more unique antipsychotics dispensed).

#### Demographic information

We used the BC Vital Statistics database to identify cases and to exclude those with schizophrenia who died in the 30 days following the EHE from the control group. The age, sex, and the geographic health region of residence^29^ for cases were extracted from the Vital Statistics database. The age, sex, and the health region of residence for controls were extracted from the BC Client Roster, which captures all individuals registered with MSP. In addition to excluding those who died within 30 days of the EHE, we excluded those missing information on age, sex, or health region of residence from the control group.

The province of BC has several pharmacare plans to help MSP enrollees cover the cost of prescriptions and medical supplies^30^. Plan C covers 100% of prescription drug costs for enrollees receiving income assistance through the Ministry of Social Development and Poverty Reduction. Individuals are enrolled in Plan C based on family situation, income, assets, and benefits. We created a binary variable for income assistance indicating whether any prescription was dispensed to a case or control under Plan C in the year preceding the EHE.

#### Healthcare usage indicators of schizophrenia severity

To characterize schizophrenia severity, we extracted the following variables for the two years preceding the EHE (i.e., 25 June 2019 to 24 June 2021): (1) number of outpatient visits related to schizophrenia; (2) number of hospital admissions related to schizophrenia; and (3) whether or not an individual was seen at an emergency department (ED) for a mental health-related presenting complaint. Records with a primary diagnostic code for schizophrenia were extracted from MSP outpatient physician billings and the hospital discharge abstract database (DAD). MSP billings were identified using the International Classification of Diseases, 9th Edition (ICD-9) code 295. DAD records were classified using diagnostic short code 056, based on the ICD-10. The National Ambulatory Care Reporting System (NACRS) broadly characterizes presenting complaints by the symptoms or conditions for which patients seek emergency care. We identified all records with mental health-related presenting complaint codes 351–359.

### Statistical analyses

#### Primary analysis

We used multiple logistic regression to estimate the association between mortality during the EHE and antipsychotic dispensations in the 30 days prior. Models were adjusted for 12 covariates with the potential to confound the antipsychotic dispensations and EHE mortality, including age, sex, health region of residence, time since schizophrenia CDR entry, income assistance, comorbidities (diabetes, hypertension, and substance use disorder), and indicators of schizophrenia severity (outpatient visits, hospital admissions, ED visits, and relapse in the past year). We first used simple logistic regression to individually assess the relationship between each covariate and EHE mortality. We then built an adjusted multiple regression model for antipsychotic dispensations including all twelve of the covariates as potential confounders (Eq. 1).

1 $$EHE\:Mortality\:=Any\:antipsychotic\:dispensation+Age+Sex+\dots\:+Schizophrenia\:relapse\:$$

#### Follow-up analyses

To further investigate the effect of antipsychotic medications, we used four other definitions of exposure to refit the fully adjusted model (Eq. 1) by replacing the dispensation of any antipsychotic with the following indicators: (1) continuous dispensation of antipsychotics in the year preceding the EHE (yes/no) (Eq. 2); (2) combination therapy in the 30-day window before the EHE (no antipsychotic/monotherapy/combination therapy) (Eq. 3); (3) any injectable antipsychotic dispensed in the 30-day window (yes/no) (Eq. 4), and; (4) dispensations of each individual antipsychotic drug (yes/no) adjusted for all other antipsychotics in the 30-day window, where there were at least five dispensations among both cases and controls (Eq. 5). All analyses were conducted using R v.4.3.1^31^.

2 $$EHE\:Mortality\:=\:Continuous\:antipsychotic\:dispensation+Age+Sex+\dots\:+Schizophrenia\:relapse$$

3 $$EHE\:Mortality\:=\:Combination\:therapy+Age+Sex+\dots\:+Schizophrenia\:relapse$$

4 $$EHE\:Mortality\:=\:Injectable\:antipsychotic\:dispensation+\:Age+Sex+\dots\:+Schizophrenia\:relapse$$

5 $$EHE\:Mortality={Antipsychotic\:drug}_{1}+\dots\:+\:{Antipsychotic\:drug}_{n}\:+Age+Sex+\dots\:+Schizophrenia\:relapse$$

## Results

### Description

There were 68,582 people in the BC schizophrenia CDR at the start of the 2021 EHE. Of those, 137 died during the EHE and were defined as cases. Among the remaining 68,445 people with schizophrenia, 57,394 were retained as controls after the removal of individuals who entered the schizophrenia CDR within 30 days of the start of the EHE (*N* = 2,825), were missing demographic data or did not reside in the province (*N* = 8,097), or died within the 30 days following the EHE (*N* = 129). Overall, cases were older than controls by an average of 13 years, with a higher proportion of males (62% male vs. 57% female). More cases (82%) had been dispensed at least one antipsychotic medication in the 30 days before the EHE compared with controls (56%). Consistent with population distributions, most cases and controls resided in the Fraser and Vancouver Coastal Health regions that cover the greater Vancouver area. On average, cases used more schizophrenia and mental health services than controls in the two years before the EHE. They had more frequent physician visits (14 vs. 7 visits), hospital admissions (0.45 vs. 0.22 admissions), and were more likely to present to the ED for mental health complaints (26% vs. 19%). Cases were also in the schizophrenia registry for longer (18 vs. 13 years), were more likely to receive income assistance through Plan C (65% vs. 44%) and had a higher prevalence of diabetes (38% vs. 20%) and hypertension (48% vs. 26%), but not substance use disorder (42% vs. 43%) (Table 1). The most common cause of death in the cases was exposure to excessive natural heat (49.6%), followed by diseases of the circulatory system (11.7%) and neuropsychiatric conditions (10.9%) (Table 2).

**Table 1:** Descriptive statistics for study cases who died during the 2021 extreme heat event (EHE) and surviving controls. Percentages are the proportion of cases and controls in each category. Most antipsychotic variables reflect dispensation in the 30 days prior to the 2021 EHE, but continuous dispensation was defined as covering at least 75% of the year prior to the EHE.

	Cases (*N* = 137)	Controls (*N* = 57,394)
Antipsychotic dispensations		
Any dispensation	82%	56%
Continuous dispensation of any antipsychotic	75%	49%
Injectable	21%	13%
1 antipsychotic dispensation	36%	37%
2 + antipsychotic dispensations	47%	19%
Medication		
Aripiprazole	7.0%	12%
Clozapine	17%	6.7%
Flupentixol	4.4%	1.2%
Haloperidol	5.8%	1.1%
Lithium	10%	5.3%
Loxapine	7.3%	2.8%
Methotrimeprazine	3.6%	1.1%
Olanzapine	20%	12%
Paliperidone	10%	7.6%
Quetiapine	22%	14%
Risperidone	18%	10%
Zuclopenthixol	5.8%	2.0%
Anticholinergic dispensation		
Covariates		
Mean age (SD)	63 (13)	50 (17)
Male sex	62%	57%
Plan C Income Assistance	65%	44%
Mean years since registry entry (SD)	18 (9)	13 (9)
Schizophrenia relapse in the year before the EHE	45%	29%
Mean inpatient admissions (SD) two years before EHE	0.45 (1.46)	0.22 (0.78)
Mean outpatient visits (SD) two years before EHE	14 (27)	7 (19)
Mental health ED visit two years before EHE	26%	19%
Diabetes	38%	20%
Hypertension	48%	26%
Substance use disorder	42%	43%
Health region of residence		
Fraser	36%	20%
Interior	15%	12%
Northern	2.9%	6.5%
Vancouver Coastal	31%	37%
Vancouver Island	15%	24%

**Table 2:** Cause of death for cases (*N* = 137) as described in vital statistics.

Cause of death	Proportion of deaths (%)
Exposure to excessive natural heat	49.6
Diseases of the circulatory system	11.7
Neuropsychiatric conditions	10.9
Other ill-defined and unspecified causes of mortality	9.5
Neoplasms	7.3
Diseases of the respiratory system	3.6
Other causes	3.6
Diabetes mellitus	2.2
Chronic liver disease and cirrhosis	1.5

### Antipsychotic dispensations and EHE mortality

#### Primary analysis

**Fig. 1:**
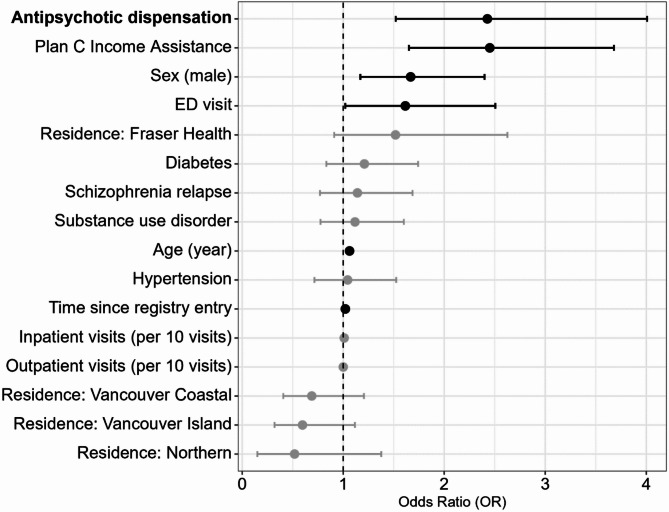
Adjusted odds ratios (ORs) for primary analysis (Eq. 1) of the association between antipsychotic dispensation and extreme heat event mortality among people with schizophrenia. ORs are shown ranked by point estimate with statistically significant associations shown in black for clarity. The referent health region of residence is the Interior region.

In the fully adjusted model (Eq. 1), the odds ratio (OR) [95% confidence interval] for dispensation of any antipsychotic medication in the 30-day window before the EHE was 2.43 [1.52, 4.01]. In this model, age, sex, income assistance, ED visits, and time since registry entry were also significantly associated with EHE mortality (Fig. 1). The OR for income assistance was similar to that for any antipsychotic dispensation, at 2.45 [1.65, 3.68]. When compared with residing in the Interior health region, the OR for residing in the Fraser health region was 1.52 [0.15, 1.38]), while residing in all other health regions was associated with decreased EHE mortality, although these effects were not significant at the *p* < 0.05 level. The ORs for comorbidities, schizophrenia relapse, the number of schizophrenia-related inpatient and outpatient visits were not significant at the *p* < 0.05 level.

#### Follow-up analyses

**Fig. 2:**
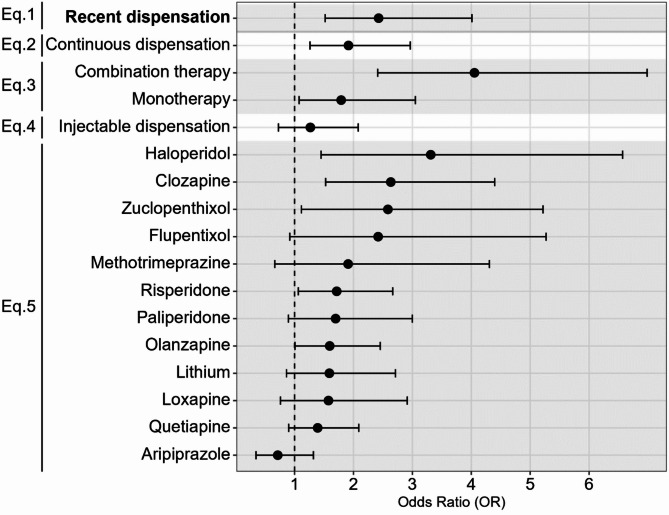
Adjusted ORs for follow-up analyses (Eqs. 2–5) of the association between antipsychotic dispensation and extreme heat event mortality among people with schizophrenia. All ORs are adjusted for age, sex, health region of residence, Plan C income assistance, time since registry entry, inpatient and outpatient visits for schizophrenia, mental health ED visits, diabetes, hypertension, substance use disorder, and schizophrenia relapse.

Continuous dispensation of any antipsychotic (> 274 days) in the year preceding the EHE (Eq. 2) was significantly associated with EHE mortality (OR = 1.92 [1.26, 2.96]) (Fig. 2). Combination therapy (Eq. 3) was associated with higher odds of EHE mortality (OR = 4.05 [2.41, 6.98]) than monotherapy (OR = 1.79 [1.08, 3.05]), both compared with no antipsychotic therapy (Fig. 2). Dispensations of injectable antipsychotics (Eq. 4) were not associated with EHE mortality after adjustment for all other covariates (Fig. 2).

Finally, some individual antipsychotics had much larger effects than others (Eq. 5). After adjusting for all other covariates, the ORs were 3.31 [1.45, 6.57] for haloperidol, 2.63 [1.53, 4.40] for clozapine, 2.58 [1.12, 5.22] for zuclopenthixol, 1.71 [1.06, 2.67] for risperidone, and 1.60 [1.01, 2.45] for olanzapine (Fig. 2). Except for aripiprazole, the ORs for dispensations of most other antipsychotics were also elevated but insignificant at *p* < 0.05. The other covariates in the drug-specific models did not change substantially from the primary model (Fig. 1).

## Discussion

In the primary analysis we found that the odds of death among people with schizophrenia during the 2021 EHE in BC were higher for those who had been dispensed any antipsychotic within the preceding 30 days. In addition to the effect of antipsychotics, we identified several other factors associated with EHE risk in this population. First, enrolment in the provincial income assistance program had the largest effect on mortality during the EHE (OR = 2.45 [1.65, 3.68], which was similar in magnitude to the effect for any antipsychotic dispensation. We also found that EHE mortality was significantly elevated for people who were older (OR = 1.06 per year [1.05, 1.08]), male (OR = 1.67 [1.17, 2.40]), had been seen in the ED for a mental health-related presenting complaint (OR = 1.61 [1.02, 2.51]), and had been in the schizophrenia registry for longer (OR = 1.02 per year [1.00, 1.04]).

Follow-up analysis showed those continuously dispensed antipsychotics in the year prior to the EHE (> 75% of the year) were also at higher risk (OR = 1.92 [1.26, 2.96]), though lower than that for the 30-day window (OR = 2.43) [1.52, 4.01]. We observed a marked increase in EHE mortality associated with antipsychotic combination therapy, with individuals dispensed two or more antipsychotics concomitantly having four times the odds of death compared with individuals who were not dispensed antipsychotics. We found that there was no increased risk from dispensation of an injectable antipsychotic. Haloperidol, clozapine, zuclopenthixol, risperidone, and olanzapine were significantly associated with increased EHE mortality, and risk for most other antipsychotics was elevated.

### Antipsychotics and heat mortality

Our findings are consistent with other studies that have reported an association between antipsychotic medications and mortality during extreme heat^12,32–37^. For example, a meta-analysis of risk factors for deaths during heat waves reported a pooled OR of 1.9 [1.3, 2.8] across four case-control studies for people taking psychotropic medications^36^ which includes antipsychotics. Similarly, during the 2003 European heat wave, antipsychotic medications were associated with an OR of 2.09 [1.89, 2.35] when comparing deaths among adults aged 70–100 years with matched surviving controls^37^. However, previous reports were either unable to disentangle the effect of antipsychotic medications from the impacts of the illnesses they were used to treat, or they did not examine the effect of antipsychotic medication among people with specific illnesses such as schizophrenia.

Although the exact mechanisms by which antipsychotics impact the risk of heat-related death is unclear, they are known to affect thermoregulation. For example, medications with anticholinergic properties inhibit the binding of the neurotransmitter acetylcholine to its receptor^38^. This has important implications for thermoregulation because acetylcholine must bind to its receptor to activate sweat glands, which are the primary mechanism by which the body cools itself^39^. Antipsychotics can also affect the neurotransmitters dopamine, serotonin, and noradrenaline, which each have a function in thermoregulation, though their exact roles are not completely understood^38^.

Our study provides evidence that antipsychotics were independently associated with death during the 2021 EHE among people with schizophrenia. We found that the OR for any antipsychotic dispensation in the 30-day window before the event was 2.43 [1.52, 4.01], even after controlling for several other important factors. We also found the effect was somewhat higher for individuals with more recent exposure (30-day window before the EHE) than for those who had continuous exposure in the preceding year (OR = 1.92 [1.26, 2.92]), though the confidence intervals were overlapping.

The risks associated with antipsychotic medications varied by the number and type of drugs dispensed. There was no increased risk associated with dispensation of an injectable antipsychotic, which require frequent healthcare contact to administer. This increased level of monitoring prior to the EHE could have been protective, or slower release formulations may be lower risk. Our results suggest that the adverse effects of antipsychotics may be amplified when used together in combination therapy. We found that the odds of death were 4.05 [2.41, 6.98] for individuals dispensed two or more types of antipsychotic medication and 1.79 [1.08, 3.05] for individuals dispensed only one antipsychotic, both compared with no dispensation of an antipsychotic.

Antipsychotic combination therapy often involves the use of two or more drugs that act on different neurotransmitter pathways and are prescribed together to treat acute schizophrenia symptoms and/or schizophrenia that is unresponsive to a single drug^28^. Antipsychotic combination therapy is common in clinical practice globally with rates of 10–20% among outpatients and up to 40% among inpatients^40^. A study examining interactions between different antipsychotics prescribed to individuals with schizophrenia found that 23% of participants encountered harmful effects of combined therapies^41^. The increased risk we report for combination therapy may be due to different medications targeting different thermoregulation pathways, the adverse effects from drug-drug interactions, higher schizophrenia severity, or a combination of these factors.

Nearly all (11 of 12) of the antipsychotic medications were associated with elevated EHE mortality. Antipsychotic medications are classified as typical (first generation) or atypical (second generation). Generally, typical antipsychotics work by blocking dopamine receptors and are more likely to cause extrapyramidal side effects (EPS), a serious adverse outcome^27^. In addition to the anticholinergic properties of typical antipsychotics themselves, anticholinergic medications are often prescribed to treat EPS symptoms caused by antipsychotics^42^. Atypical antipsychotics act mostly on the serotonin system and come with a reduced risk of EPS^43^. Apart from clozapine, the typical antipsychotics in our study were associated with the highest EHE mortality: haloperidol (OR = 3.31 [1.45, 6.57]), zuclopenthixol (OR = 2.58 [1.12, 5.22]), flupentixol (OR = 2.42 [0.92, 5.27]), and methotrimeprazine (1.91 = [0.67, 4.31]).

Although haloperidol has a weak anticholinergic profile on its own, it is a high potency typical antipsychotic that commonly causes serious adverse effects including EPS^42^. Anticholinergics are often co-prescribed with haloperidol, as well as zuclopenthixol. A study by Khaja et al.^44^ examining patterns of antipsychotic prescription in psychiatric hospital outpatients found that anticholinergics were co-prescribed at a rate of 71.2% for haloperidol and 88.1% for zuclopenthixol. Clozapine is an atypical antipsychotic prescribed to individuals with treatment-resistant schizophrenia^42,43,45^ and has the highest anticholinergic properties of all antipsychotics dispensed in this population^38–40^. It is also often used in combination therapy^46^. Therefore, clozapine users may be at particular risk during EHEs because of potentially greater disease severity as well as its anticholinergic potency.

### Other risk factors

Beyond antipsychotics, we found that several other variables were associated with increased odds of EHE mortality, highlighting that the risks for people with schizophrenia are likely due to complex and overlapping factors. First, being enrolled in the provincial income assistance program through Plan C had the highest OR, indicating that low income was an important risk factor, even after adjusting for antipsychotics and all other variables. This suggests that the social determinants of health, such as poverty, housing quality, and access to air conditioning, may play a key role in heat-related risks for people with schizophrenia^16,47^. Our findings also align with other reports that older age and male sex are risk factors for mortality among people with mental illnesses during heat events^7,48^. Older populations are also simultaneously more susceptible to the impacts of extreme heat and the anticholinergic effects of antipsychotic medications^42^. Indicators of schizophrenia severity were also associated with increased odds of EHE mortality, including having presented to the ED in the past year for a mental health complaint and having lived longer with the condition.

### Strengths and limitations

The biggest strength of this study was our ability to comprehensively compare risk factors among the entire population of individuals with schizophrenia in BC. In particular, the PharmaNet database allowed access to the entire dispensation history of all cases and controls. In contrast, previous research on the links between mental illness and heat-related mortality has been limited to comparing risks in the general population and has not been able to isolate risk factors for those with a specific condition. Additionally, the provincial COVID-19 Data Library enabled us to link individual-level data to include multiple health-related variables. For example, we were able to assess individual-level data on income assistance rather than using area-level indicators.

Our study also has important limitations. First, the study is predicated on an administrative definition of schizophrenia, not a clinical diagnosis. However, a similar algorithm for detecting individuals with chronic psychotic illness using linked health administrative databases was validated in Ontario, Canada, with sensitivity ranging from 90.1–98.8%^49^. Second, data on antipsychotic medications was compiled from records on drug dispensations from community pharmacies, a proxy that most likely overestimates exposure because we could not determine whether individuals took their dispensed medications as prescribed. Lastly, the clinical indication, or the severity of schizophrenia in an individual, influences the likelihood of receiving treatment^50^ and may explain some of the association between antipsychotic treatment and EHE mortality. However, we identified antipsychotic dispensation as an independent risk factor for death among people with schizophrenia before and after controlling for important confounding factors such as age, sex, income assistance, multiple comorbidities, and multiple indicators of disease severity.

## Conclusions

Overall, our study confirms that antipsychotic medication is an important risk factor for EHE mortality among people with schizophrenia, and highlights the increased risk associated with combination therapy. However, we also report that antipsychotic medications are just one factor among complex and inter-related factors that influence susceptibility, including age, sex, low income, and higher disease severity. In addition to the risk factors identified in this study, the BC Coroner’s service reported that most people who died during the 2021 EHE died inside of their homes and lived alone^51^.

Antipsychotic medications are a lifesaving therapy for people with schizophrenia^52^ and our analysis has not compared the risks of heat-related injury to this substantial benefit. Our results are not clinical and cannot be used to assess whether antipsychotic therapy should be discontinued or modified during hot weather. However, more than half of the cases in this study sought primary care for a schizophrenia-related reason in the 30-day window before the EHE, and more than 80% were dispensed an antipsychotic. These contacts with the healthcare system provide excellent opportunities for clinicians and pharmacists to provide more information about antipsychotics and heat-related risks to people with schizophrenia and those who support them.

Our results also highlight that interventions should target other risk factors in this population, such as economic marginalization and social isolation. Examples include working with partners who support people with schizophrenia and their care networks^53^ programs that make mechanical cooling technologies available^54^ and routine health checks during hot weather events^53,55^. Indeed, prior work from our group has called for interventions that increase social connection as a key public health response to EHEs^56^.

## Supplementary Information

Below is the link to the electronic supplementary material.

Supplementary Material 1

## Data Availability

The data supporting this study are available from the British Columbia Ministry of Health to support public health response and are not accessible to the public or research community. Requirements and mechanisms for accessing the data vary between BC government ministries, health sector partners, researchers, and other organizations or individuals. Use is governed by legislation, government policies, professional codes of ethics, and standards of practice. For detailed information on how to request access to this data see https://www2.gov.bc.ca/gov/content/health/conducting-health-research-evaluation/data-access-health-data-central.
