# Supplementary material for: Antipsychotics and other risk factors for mortality among people with schizophrenia during an extreme heat event: a population-based case-control study
Source: Sci Rep. 2025 Oct 3;15:34505. doi: 10.1038/s41598-025-17591-0 (PMC12494884; doi:10.1038/s41598-025-17591-0)
Supplement: Supplementary file 1 — Supplementary Material 1 [file 41598_2025_17591_MOESM1_ESM.pdf]

Antipsychotics and other risk factors for mortality among people with schizophrenia during an extreme heat event: A population-based case-control study

Shirley X. Chen[1,2*], Michael J. Lee[1], David A. McVea[1], Sarah B. Henderson[1]

[1] Environmental Health Services, British Columbia Centre for Disease Control, Vancouver, Canada
[2] Public Health Agency of Canada, 100 Colonnade Rd, Ottawa, Canada
[*]shirley.chen@bccdc.ca

Supplementary Table 1. Description of the primary study variables and their sources.

Variable	Type	Source
Extreme heat event (EHE) mortality	Binary variable (case/control) indicating death during the event or survival by 30 or more days	Vital Statistics
Antipsychotic dispensation	Binary variable (yes/no) indicating any dispensation of antipsychotic medication	PharmaNET
Age	Integer (years)	Cases: Vital Statistics<br>Control: Client Roster
Sex	Dichotomous (male/female)	Cases: Vital Statistics<br>Control: Client Roster
Health region of residence	Categorical variable with five health regions (region)	Cases: Vital Statistics<br>Control: Client Roster
Income assistance (Plan C)	Binary variable (yes/no) indicating whether any prescription had been dispensed under the Plan C income assistance program in the 90 days prior to the EHE	PharmaNET
Time since registry entry	Integer (years)	Schizophrenia Chronic Disease Registry
Schizophrenia relapse	Binary (yes/no)	Schizophrenia Chronic Disease Registry
Outpatient visits for schizophrenia	Count	Medical Services Plan database
Hospital admissions for schizophrenia	Count	Discharge Abstract Database
ED visit for mental health	Binary (yes/no)	National Ambulatory Care Reporting System
Diabetes	Binary (yes/no)	Diabetes Chronic Disease Registry
Hypertension	Binary (yes/no)	Hypertension Chronic Disease Registry
Substance use disorder	Binary (yes/no)	Substance use disorder Chronic Disease Registry

Supplementary Table 2. Cause of death for all cases from BC Vital Statistics

**Cause of Death**	**Proportion (%)**
Exposure to excessive natural heat	49.6%
Diseases of the circulatory system	11.7%
Neuropsychiatric conditions	10.9%
Other ill-defined and unspecified causes of mortality	9.5%
Neoplasms	7.3%
Diseases of the respiratory system	3.6%
Other causes	3.6%
Diabetes mellitus	2.2%
Chronic liver disease and cirrhosis	1.5%

Supplementary Table 3. Univariate and adjusted multivariable logistic regression results

**Variables**	OR (95% CI)	
**Antipsychotic Dispensations**	**Crude**	**Adjusted**
Any antipsychotic dispensation	3.76** (2.47, 5.99)	2.43** (1.52, 4.01)
Continuous antipsychotic dispensation	3.15** (2.16, 4.71)	1.92** (1.26, 2.96)
Injectable antipsychotic dispensation	1.84** (1.20, 2.73)	1.53 (0.96, 2.38)
1 antipsychotic dispensation	2.46** (1.53, 4.08)	1.79* (1.08, 3.05)
2+ antipsychotic dispensations	6.31** (4.00, 10.3)	4.05** (2.41, 6.98)
**Antipsychotic Drugs**		
Aripiprazole	0.60 (0.30, 1.09)	0.72 (0.35, 1.32)
Clozapine	2.80** (1.74, 4.30)	2.63** (1.53, 4.40)
Flupentixol	3.85** (1.50, 8.02)	2.42 (0.92, 5.27)
Haloperidol	5.56** (2.49, 10.7)	3.31** (1.45, 6.57)
Lithium	2.05* (1.13, 3.44)	1.59 (0.86, 2.71)
Loxapine	2.71** (1.33, 4.91)	1.58 (0.76, 2.91)
Methotrimeprazine	3.41** (1.20, 7.52)	1.91 (0.67, 4.31)
Olanzapine	1.96** (1.27, 2.93)	1.60* (1.01, 2.45)
Paliperidone	1.39 (0.77, 2.34)	1.70 (0.90, 3.00)
Quetiapine	1.67* (1.10, 2.48)	1.39 (0.90, 2.09)
Risperidone	1.96** (1.24, 2.97)	1.71* (1.06, 2.67)
Zuclopenthixol	3.09** (1.39, 5.92)	2.58* (1.12, 5.22)
**Covariates**		
Age	1.05** (1.04, 1.06)	1.06** (1.05, 1.08)
Sex (male)	1.24 (0.88, 1.76)	1.67 ** (1.17, 2.40)

Plan C Income Assistance	2.44** (1.73, 3.48)	2.45** (1.65, 3.68)
Time since CDR registry entry (years)	1.06 (1.04, 1.09)	1.02* (1.00, 1.04)
Schizophrenia relapse in year before EHE	2.03** (1.45, 2.85)	1.14 (0.77, 1.69)
Number of inpatient admissions in two years before EHE	1.21** (1.06, 1.33)	1.01 (0.94, 1.25)
Number of outpatient visits in two years before EHE	1.01** (1.00, 1.01)	1.00 (1.00, 1.00)
Mental health ED visit in two years before EHE (yes/no)	1.42 (0.95, 2.06)	1.61* (1.02, 2.51)
Diabetes	2.44** (1.72, 3.44)	1.21 (0.83, 1.74)
Hypertension	2.62** (1.87, 3.66)	1.04 (0.72, 1.52)
Substance use disorder	0.97 (0.69, 1.35)	1.12 (0.78, 1.60)
Health region of residence		
Interior	Ref	Ref
Fraser	1.47 (0.89, 2.52)	1.52 (0.91, 2.63)
Northern	0.37 (0.11, 0.98)	0.52 (0.15, 1.38)
Vancouver Coastal	0.68 (0.41, 1.20)	0.69 (0.41, 1.21)
Vancouver Island	0.52 (0.28, 0.96)	0.60 (0.32, 1.12)

**p<0.05*; ***p<0.01*
